# Latent profiles of mental health in older adults living in nursing homes: the challenge of suicide prevention

**DOI:** 10.3389/fpsyg.2025.1740402

**Published:** 2026-01-07

**Authors:** Alicia Sales, Rita Redondo, Carolina Pinazo-Clapés, Sacramento Pinazo-Hernandis, Josep Pons, Irene Checa

**Affiliations:** 1 Department of Developmental Psychology, Universitat de Valencia, Valencia, Spain; 2 Universidad Europea de Valencia, Faculty of Health Sciences, Department of Psychology, Valencia, Spain; 3 Department of Social Psychology, Universitat de Valencia, Valencia, Spain

**Keywords:** latent profile analysis, mental health, nursing homes, older adults, risk and protective factors, suicidal ideation

## Abstract

**Objectives:**

Suicide prevention in nursing homes requires a deeper understanding of the psychological mechanisms underlying suicidal ideation. This study aimed to identify mental health profiles in institutionalized older adults based on risk and protective variables, and to explore their association with suicidal ideation.

**Methods:**

A total of 231 older adults (60–97 years) from nine Spanish nursing homes were assessed on depression, hopelessness, perceived burden, purpose in life, resilience, and self-efficacy. Latent Profile Analysis (LPA) was used to identify distinct profiles, and ANCOVA tested differences in suicidal ideation across groups.

**Results:**

Four psychological profiles were identified: (1) High Risk (high symptomatology, low protection), (2) Burdensomeness (low depression and hopelessness, high burden), (3) Weakened Strengths (low symptomatology, low resources), and (4) Optimal Mental Health (low risk, high protection). Suicidal ideation levels differed significantly across profiles, and these differences remained after controlling for age, sex, and perceived health. The High Risk group showed the highest levels of suicidal ideation, whereas the Optimal Mental Health group showed the lowest.

**Conclusion:**

These profiles offer a basis for more personalized and effective prevention interventions tailored to each group’s risk-protection balance. Screening for suicidal ideation in nursing homes should incorporate both risk factors (depression, hopelessness, perceived burden) and protective factors (resilience, purpose in life, self-efficacy). A person-centered approach allows gerontologists to tailor prevention strategies to specific psychological profiles.

## 1 Introduction

Suicide represents a significant public health challenge worldwide, claiming the lives of over 700,000 individuals each year (WHO, 2024). Among these, older adults consistently present some of the highest suicide rates globally (Chauliac et al., 2020; De Leo, 2022). Despite this, research on suicide in late life remains scarce, especially among institutionalized people (Ki et al., 2024; Pinazo-Clapés et al., 2025).

The phenomenon of suicide in nursing homes represents a complex and under-researched phenomenon (Chauliac et al., 2020; Liu et al., 2024). Suicidal behaviour, which encompasses suicidal ideation on the one hand and suicidal action on the other, occurs more frequently in people suffering from depression or anxiety (43%) and in those living in institutionalised settings (19.2%) (Bareeqa et al., 2023; Beach et al., 2021; Salvatore, 2023). However, the scientific literature on suicidal behaviour in settings such as nursing homes remains limited (Connors et al., 2025; Ki et al., 2024; Liu et al., 2024).

A plethora of theories have been postulated in an attempt to enhance our comprehension of the factors associated with suicidal behaviour. It has been posited that a number of mechanisms could be transferred to older people, with a particular focus on those residing in nursing homes. However, none of these theories has been specifically designed to explain the phenomenon of suicide at this stage of the life cycle. In this regard, variables such as perceived burden, depression, and hopelessness, assume a particularly salient role in older adults living in long-term care facilities.

According to the interpersonal-psychological theory of suicide (IPTS; Klonsky and May, 2015), two interpersonal aspects that explain suicidal ideation are frustrated belonging and perceived burden. Recent studies support that, within the IPTS model, perceived burden is the factor with the strongest empirical support in predicting suicidal ideation, even above the feeling of frustrated belonging, where its effect appears to be smaller compared to perceived burden (Keefner and Stenvig, 2020; Shim et al., 2021; Segal et al., 2025). Furthermore, this greater effect of perceived burden is especially evident in older people, particularly those with illnesses that limit their daily lives, a common factor in most older people living in nursing homes (Bickford et al., 2021; Cukrowicz et al., 2011; Khazem et al., 2015). However, research on this perception in older people living in residential care is still limited, especially in contrast to the evidence available for older adults living in community settings.

Conversely, hopelessness plays a pivotal role in contemporary suicide theories. The IPTS theory, the Three Step Theory (3ST) and the Integrated Volitional Motivational Model (Klonsky and May, 2015; O’Connor and Kirtley, 2018; Van Orden et al., 2010) all suggest, either directly or indirectly, that hopelessness is one of the main precursors of suicidal thoughts that can lead to suicidal behaviour (Beach et al., 2021; Klonsky et al., 2018). Indeed, hopelessness has even been identified as a better predictor of suicidal behaviour than depression (Hernandez et al., 2021). Although depression is not mentioned in any of the above theories, it is a relevant variable to explore in older populations as it has been reported as a significant risk factor (Segal et al., 2025; Zhang et al., 2021; Yang et al., 2021) in both community-dwelling older adults and those in nursing homes (Bernier et al., 2020; Malfent et al., 2010; Nie et al., 2020).

However, while focusing on risk variables has been the norm in recent literature, this strategy is insufficient for a preventive and interventional approach (Cramer and Tucker, 2021; Hawton et al., 2022). Although recent studies are beginning to expand the available evidence, research on protective factors against suicidal behaviour in the older population remains limited (Ki et al., 2024). In this regard, life purpose is one of the most frequently reported variables with a clear protective effect against suicidal ideation in older people (Heisel et al., 2020; Ki et al., 2024).

Another widely supported variable is resilience. Several studies with older adults have consistently demonstrated a significant inverse relationship with suicidal behaviour (Liu et al., 2024; Yang et al., 2021; Liu et al., 2024; Zhang et al., 2021). While, again, research on the protective role of resilience has emphasised older people living in the community, few studies have focused on the role it may play in people living in residential care (Yang et al., 2021; Liu et al., 2024).

A variable related to resilience that has a relevant role as a possible protective factor is self-efficacy, as it is a key element in problem solving in the face of stressful events and acts as an element of resilience against suicidal ideation in older people by promoting more active and solution-oriented coping strategies (Malfent et al., 2010; Vilhjálmsson et al., 1998). Thus, self-efficacy may act as a mediator between social support and suicidal ideation (Olatunji et al., 2020). According to this perspective, perceived control over one’s actions influences decisions and perseverance in the face of difficulties. Self-efficacy has been shown to play a key role in mental health and psychological adjustment (Olatunji et al., 2020).

Thus, exploring the most relevant risk factors (such as depression, hopelessness, and perceived burden) and protective factors (such as meaning in life, resilience, and self-efficacy) simultaneously can help us to understand the different mental health profiles of older adults living in nursing homes. These profiles could then be used as strategic tools for the proactive prevention of suicidal thoughts, helping to plan interventions tailored to each individual’s needs and implement programmes aimed at promoting mental health in older people (Connors et al., 2025).

### 1.1 Present study

In order to address suicide prevention and mental health care in older people living in long-term care facilities, it is essential to deepen the understanding of suicidal ideation and its manifestation in this context. However, most studies have addressed the phenomenon from a predictive variable’s perspective, but very few with a person-oriented approach like Latent Profile Analysis (LPA). As Lee (2023) points out, since the causal relationship between suicidal ideation and suicide has not yet been fully established, detailed analyses are needed to identify target groups and thus design more effective prevention and early intervention strategies. Only one study has used latent profile analysis to identify health profiles in older people living in nursing homes (Yuan et al., 2022) but they only take into account self-reported suicidal ideation at admission, not during the stay.

In this sense, the present study applies a LPA to characterise different clinical profiles of suicidal ideation in this population. LPA is a person-centered approach that identifies unobserved subgroups based on individuals’ response patterns across multiple observed variables, thus focusing on configurations of characteristics rather than on isolated variables. As Ferguson et al. (2020) point out, LPA has three characteristics: individual differences are important and must be taken into account in the explanation of a phenomenon; these differences occur within a logic that allows grouping into patterns or profiles; and finally, these profiles are significant and occur in different individuals. Therefore, the aim of this article is to explore the existence of mental health profiles in nursing homes so that appropriate detection and prevention programmes can be designed.

## 2 Method

### 2.1 Participants

A total of 231 older adults aged 60–97 years (*M* = 78.99, SD = 8.83) participated in the study, including 95 men (41.1%) and 136 women (58.9%). Participants resided in facilities that provide long-term care and assistance to older adults, generally in a situation of physical or cognitive dependence or in a context of social vulnerability. From a pull of 30 residential centres, 9 were randomly selected, and from these, those who met the study’s inclusion criteria were invited to apply. Approximately 25–30 residents took part from each participating centre.

The inclusion criteria for participation in the study were: Being 60 years of age or older and having preserved cognitive abilities or presenting only mild cognitive impairment (MCI), that is, obtaining scores above 19 on the Mini-Mental State Examination (MMSE), in its adapted Spanish version (Mini-Mental Cognitive, MEC) (Lobo et al., 1999). This criterion aimed to ensure that all participants had sufficient cognitive capacity to provide informed consent and to reliably complete the self-report instruments. The mean MMSE score was 29.17 (SD = 3.99), with participants with MCI representing 16.4% of the total sample. Participants with MCI were included because they were able to respond to all questionnaires during individualized interviews conducted by trained staff. Including this group ensures that the sample reflects the institutionalized population and allows for the capture of emotional and psychological aspects related to these participants.

Exclusion criteria: Having suffered an acute illness or hospitalisation or unstable chronic illness in the last month or having a serious illness; or having a diagnosis of mental illness in an acute phase or with significant psychiatric decompensation at the time of assessment, which could hinder the ability to provide informed consent or respond reliably. This evaluation was carried out by the healthcare team at each care home, including the centre’s physician and psychologist.

Scores on the Mini-Mental State Examination (MMSE) scales, and information for inclusion and exclusion criteria were extracted through the residential management software used in Spanish residential centres. This database systematically collects and stores the results of the assessments carried out by the professionals of the centres every six months.

### 2.2 Variables and instruments

#### 2.2.1 Risk factors

Suicidal ideation: The suicidal ideation dimension of the Beck Suicidal Ideation Scale (SSI; Beck et al., 1979), consisting of 9 items scored from 0 to 2, was used. The reliability of the scale in this sample was *ɑ* = 0.898.

Perceived burden: The burden dimension of the Interpersonal Needs Questionnaire (INQ; Canal-Rivero et al., 2022), with 8 items in Likert format (1–7), was used. Higher scores indicate higher perceived burden. Internal consistency was *ɑ* = 0.908.

Hopelessness: The Spanish version of the Beck Hopelessness Scale (BHS; Satorres et al., 2018) was used. This version includes 20 true/false items, with total scores ranging from 0 (no hopelessness) to 20 (high hopelessness about the future). In the present sample, the internal consistency was *ɑ* = 0.880.

Depression: The abbreviated 15-item version of the Geriatric Depression Scale (GDS-15; Yesavage et al., 1983) was used, with dichotomous response (yes/no). Higher scores reflect greater depressive symptomatology. The internal consistency of the scale was *ɑ* = 0.815.

Perceived health: A visual analogue scale (VAS) was used, ranging from 0 (very poor perceived health) to 10 (excellent perceived health). This self-reported single-item scale allows participants to express their subjective evaluation of their current health status in a simple and intuitive way.

#### 2.2.2 Protective factors

Purpose of Life: The Satisfaction and Sense of Life (SSV) subscale of the abbreviated version PIL-10 (García-Alandete et al., 2013) was applied. It consists of Likert-type items (1–7); higher scores reflect higher life purpose. Reliability was *ɑ* = 0.837.

Resilience: The Brief Resilient Coping Scale (BRCS; Sinclair and Wallston, 2004) was used, with 4 Likert-type items (1–5). It assesses resilient coping. Reliability was *ɑ* = 0.754.

Self-Efficacy: The General Self-Efficacy Scale (GSE; Baessler and Schwarcer, 1996), consisting of 10 Likert-type items (1–4), was used. Higher scores indicate higher perceived self-efficacy. The reliability of the scale in this sample was *ɑ* = 0.922.

### 2.3 Procedure

After approval of the research project by the management of the residential centres, older people living in the selected nursing homes were informed of the research objectives. Those who met the inclusion criteria and agreed to take part in the study signed an informed consent form before the questionnaires were completed. Individualised, semi-structured interviews were conducted in a room in the residential centre, ensuring a quiet and distraction-free environment. The duration was approximately 60–80 min per person. The assessors were three psychologists previously trained by the study authors. The data were then entered anonymously into an SPSS database for analysis.

Since Likert-type response scales can present difficulties for older people, especially those with mild cognitive impairment or information processing problems, a colour-coded support system was implemented. Each response option within the scales was visually represented by a progressive colour palette, assigning a different colour to each response category: from cool tones for lower intensity responses to warm tones for higher intensity responses. This was intended to facilitate the understanding of the progression of responses, optimise the accessibility of the scale for people with difficulties in verbally differentiating the options and reduce ambiguity in the interpretation of responses.

This study is part of a research project that was approved by the Ethics Committee of the University of Valencia (2024-PSILOG-3281474). All procedures performed were in accordance with the ethical principles set out in the Declaration of Helsinki. Written informed consent was obtained from all participants, who were previously informed about the objectives of the study, the confidentiality of the data and their right to withdraw at any time.

### 2.4 Data analysis

To carry out the LPA, the steps proposed by Ferguson et al. (2020) were followed. First, data cleaning and checking for missing data were performed. After this, 5 iterative models were tested, following the recommendations of Tein et al. (2013) using Mixture type and Robust Maximum Likelihood Estimator (MLR). Then, each model is compared against the previous model or models to make a decision regarding the number of latent profiles in the data using the common fit indices in LPA: Akaike’s Information Criterion (AIC), Bayesian Information Criterion (BIC), Sample-Adjusted BIC (SABIC), Lo–Mendell Ruben (LMR), and Bootstrap Likelihood Ratio Test (BLRT). Regarding BIC, SABIC, and AIC, lower values indicate a better fit; however, the lowest value is relative (Masyn, 2013). Therefore, attention should be paid to the magnitude of the difference. The LMR test and the Bootstrap Likelihood Ratio Test (BLRT) compare the current model to a model with k-1 profiles. Entropy was also considered, where values of 0.80 or higher provide evidence that the classification of individual profiles in the model occurs with minimal uncertainty (Celeux and Soromenho, 1996; Tein et al., 2013), and the percentage of the sample included in the smallest profile, which is recommended to be no lower than 5% (Ferguson et al., 2020).

After selecting the model that best fits the data, it was interpreted through the mean scores on each variable (depression, hopelessness, burden, self-efficacy, purpose of life, and resilience) for each profile. Finally, to examine whether the resulting profiles differed in suicidal ideation after adjusting for potential confounding variables, an Analysis of Covariance (ANCOVA) was conducted. Suicidal ideation was entered as the dependent variable, profile membership as the fixed factor, and age, sex and perceived health as covariates. Adjusted marginal means were calculated and pairwise comparisons were conducted using Bonferroni correction. A *p*-value < 0.05 indicated statistical significance. All analyses were conducted using Mplus 8.11 (Muthén and Muthén, 2017) and IBM SPSS 28.0.

## 3 Results

The fit indices for the 5 models tested are described in Table 1. Model 4 was retained as the best model to fit the data based on the low log-likelihood value, AIC, BIC, and SABIC values, adequate entropy value, and the smallest class containing more than 5% of the sample. Although the 5-class model presents smaller values for AIC, BIC, and SABIC, the smallest class only represents 9% of the sample and the entropy value is lower.

**Table 1:** Fit indicators for each latent profile.

Classes	Log likelihood	AIC	BIC	SABIC	Entropy	Smallest class %	LMR *p*-value	BLRT *p*-value
1	−4314.120	8652.240	8693.549	8655.516				
2	−4116.197	8270.394	8335.800	8275.581	0.861	29.3%	0.015	0.016
3	−4085.571	8169.143	8258.645	8176.240	0.833	13.8%	0.06	<0.001
**4**	**−4015.384**	**8096.768**	**8210.368**	**8105.777**	**0.892**	**9.9%**	**0.41**	**<0.001**
5	−3985.425	8050.849	8188.546	8061.768	0.858	9.0%	0.20	<0.001

In bold is the retained model. AIC, Akaike information criterion; BIC, Bayesian information criterion; SABIC, sample-size adjusted Bayesian information criterion; LMR, lagrange multiplier test; BLRT, bootstrap likelihood ratio test.

On the other hand, as shown in Table 2, the results are consistent with the theoretical model, yielding four differentiated profiles based on key psychological and emotional variables such as depression, hopelessness, burden, self-efficacy, purpose in life, and resilience. These profiles allow for a better understanding of the varying levels of psychological vulnerability among institutionalized older adults.

**Table 2:** Four-profiles model.

Variable (min-max of sample)	Profile 1 (9.9%) High risk	Profile 2 (12.55%) Burdensomeness	Profile 3 (21.21%) Weakened Personal Strengths	Profile 4 (56.27%) Optimal Mental Health
Depression (0–15)	11.0(6.07)	6.27(6.07)	8.23(6.07)	3.04(6.07)
Hopelessness (0–19)	14.54(13.60)	8.36(13.60)	11.84(13.60)	4.45(13.60)
Burden (4–28)	19.21(7.50)	17.80(7.50)	6.00(7.50)	5.17(7.50)
Self-efficacy (0–40)	12.40(55.20)	21.77(55.20)	19.86(55.20)	24.89(55.20)
Purpose of Life (5–35)	10.43(24.94)	22.32(24.94)	16.52(24.94)	25.21(24.94)
Resilience (4–20)	8.44(9.56)	13.81(9.56)	12.54(9.56)	16.12(9.56)

All SDs (in parentheses) are the same across profiles, as the variances are set to 0 in the LPA calculation.

**Figure 1:**
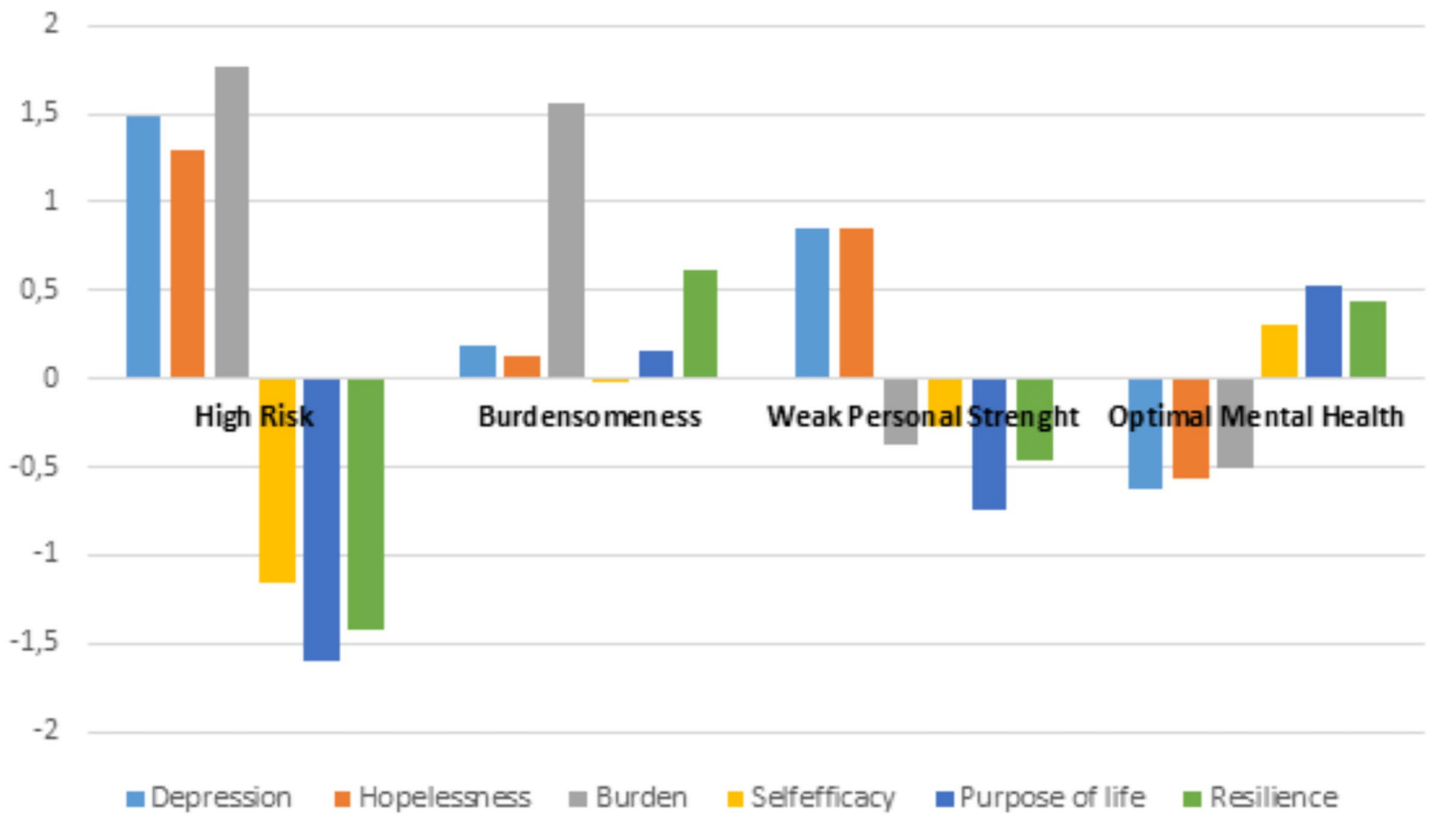
Clustered bar chart with *z*-scores for each profile in each variable.

As shown in Figure 1, Profile 1 (*n* = 23), which could be called High Risk (HR), showed high levels of hopelessness, depression, and burden, and low levels of self-efficacy, purpose in life, and resilience. Profile 2 (*n* = 29) displayed low levels of depression and retained personal strengths (self-efficacy, resilience, and purpose in life) but felt like a burden, so it could be called Burdensomeness (B). Profile 3 (*n* = 49), Weakened Personal Strengths (WPS), showed moderate depression and hopelessness, did not feel like a burden, but had low levels of purpose in life, self-efficacy, and resilience, indicating that internal strengths were significantly affected. Finally, Profile 4 (*n* = 130) showed low levels of depression, hopelessness, and burden, and high levels of self-efficacy, purpose in life, and resilience; this profile could be called Optimal Mental Health (OMH).

### 3.1 Relationship between profiles and suicidal ideation

To examine whether suicidal ideation differed across the four latent profiles after adjusting for potential confounding variables, an Analysis of Covariance (ANCOVA) was conducted with profile membership as the fixed factor and age, sex, and perceived health as covariates. The overall model was significant, *F*(9,221) = 19.64, *p* < 0.001, explaining a substantial proportion of the variance in suicidal ideation (partial η^2^ = 0.444). Among the covariates, perceived health was a significant predictor, indicating that poorer perceived health was associated with higher suicidal ideation, *F*(1,221) = 5.23, *p* = 0.023. In contrast, age was not a significant predictor, F(1,221) = 0.07, *p* = 0.795. Sex showed a significant main effect, with women reporting higher suicidal ideation than men, *F*(1,221) = 20.33, *p* < 0.001. Importantly, although women and individuals with poorer perceived health showed higher overall suicidal ideation, the pattern of differences between profiles remained consistent across these variables, with the High Risk group exhibiting the highest levels and the Optimal Mental Health group the lowest.

Crucially, the effect of latent profiles remained highly significant after covariate adjustment, *F*(3,221) = 31.02, *p* < 0.001, partial η^2^ = 0.296. Adjusted marginal means indicated that the High Risk profile showed the highest level of suicidal ideation (*M* = 7.09), followed by the Burdensomeness (*M* = 3.16) and Weakened Personal Strengths profiles (*M* = 3.02). The Optimal Mental Health profile showed the lowest levels (*M* = 0.88). Post-hoc pairwise comparisons with Bonferroni correction demonstrated that the High Risk profile differed significantly from all other profiles (*p* < 0.001), and that the Optimal Mental Health profile presented significantly lower suicidal ideation compared to Burdensomeness (*p* = 0.001) and Weakened Personal Strengths (*p* < 0.001). No significant differences were found between the Burdensomeness and Weakened Personal Strengths profiles.

## 4 Discussion

Caring for older people living in residential care homes with mental health problems poses significant challenges (Cizková et al., 2024). This study represents a novel contribution to the analysis of mental health and suicidal ideation in older people living in residential care, thanks to the methodology of analysis used, which moves away from an approach based exclusively on variables (variable-oriented approach) to adopt a person-oriented approach.

This work has sought to define different mental health profiles in older people living in nursing homes and to explore the risk and protective factors related to suicidal ideation. Four profiles were identified that offer a differentiated perspective on vulnerability and resilience, facilitating the design of intervention and prevention strategies adapted to the needs of each group. In addition, it is worth noting that the data collection was particularly rigorous, both in terms of the number and diversity of participating residences and the length and depth of each interview, which guarantees greater reliability of the data which contributes to improving the reliability of the data collected.

### 4.1 Profiles

High Risk profile is characterised by high levels of depression and hopelessness, as well as high perceived burden. Depression is one of the risk factors in older adults that has received the most scientific evidence (Beghi et al., 2021; Hernandez et al., 2021; Ki et al., 2024); Depression often coexists with other emotional and cognitive difficulties, such as feelings of uselessness or a lack of motivation. It has also been linked to an increased perception of burden, particularly among older adults in nursing homes who have functional limitations (Zhang et al., 2021). In residential settings, depression can be overlooked or mistaken for normal ageing, despite its strong predictive value for suicidal thoughts (Redondo et al., 2025). Several studies have confirmed the direct association between hopelessness and suicidal ideation in older adults, even when controlling for depression and functional impairment (Cukrowicz et al., 2011; Ribeiro et al., 2018). In this sense, hopelessness intensifies the subjective experience of suffering and, when combined with the perception of burden, forms a high-risk psychological profile.

In addition, individuals in this group show low scores on self-efficacy, resilience and purpose in life. This combination of factors indicates a marked vulnerability (Beach et al., 2021; Olatunji et al., 2020; Liu et al., 2024), which requires immediate and specialised interventions. Strategies targeting this profile should include immediate psychological care, combined with suicide prevention programmes tailored to older people, interventions focused on strengthening purpose in life and resilience, enhancing self-efficacy, and actions to improve perceived social support and reduce emotional burden (Okolie et al., 2017; Heisel and Flett, 2022; Treichler et al., 2020; Ki et al., 2024).

Within the moderate risk profiles, two subgroups are distinguished. Burdensomeness profile, which is characterised by the presence of low depressive and hopelessness symptoms together with a notable perception of burden, although these individuals retain certain levels of resilience, self-efficacy and purpose in life. This group would benefit from preventive interventions that enhance social support, encourage participation in group activities and strengthen the perception of belonging and usefulness since, as indicated by Van Orden et al. (2012), reducing their perceived burden could be an effective way to improve their well-being and quality of life. This perceived burden, which can include physical, social and emotional components (McPherson et al., 2007), is more prevalent in those living in residential care, where the need for help with daily activities is often greater (Conwell et al., 2011; Zhang et al., 2021).

On the other hand, the Weakened Personal Strengths profile shows low levels of depression, hopelessness and perceived burden, but also low purpose, resilience and self-efficacy was observed. Low purpose increases vulnerability to suicidal ideation and low resilience and self-efficacy makes them less able to cope with complexities. For this group it is essential to implement interventions that work on purpose in life, as they could be innovative ways to prevent suicidal behaviour in older adults (Heisel et al., 2020; Ostafin and Proulx, 2020).

Purpose in life is a factor that, not only prevents suicidal ideation (Beach et al., 2021; De Brigard, 2021), but also reduces stress, depression and anxiety. It is a method of coping with human problems that cause suffering and plays a mediating role in health according to longitudinal studies (Alimujiang et al., 2019; Soucase et al., 2023). In this line, the development of psychological interventions that can enhance modifiable intrapersonal factors such as resilience (Treichler et al., 2020) or coping strategies (Gysin-Maillart et al., 2020) could be innovative ways to prevent suicidal behaviour in older adults (Ki et al., 2024).

Optimal Mental Health profile, represents well-functioning individuals. Their situation highlights the importance of promoting social participation, the establishment of support networks and the consolidation of effective coping mechanisms in the older population. Health, understood as a fundamental human right, must be approached from a biopsychosocial model that recognises the dynamic interaction between biological, psychological and social factors in people’s well-being (WHO, 2024). This conception is aligned with the notion of active and healthy ageing promoted by the WHO, which seeks not only the absence of disease, but full participation in social, emotional and community life (WHO, 2020). In this sense, this profile reflects not only an absence of risk factors, but also the presence of protective factors, such as self-efficacy, resilience and purpose in life, that allow older people to face the challenges of ageing from a position of autonomy and dignity. This result challenges ageist conceptions that associate institutionalisation with situations of dependency and deterioration (Xu et al., 2022), recalling that not all older people living in residential care homes feel a burden, nor do they live their old age from emotional discomfort. This profile therefore posits a form of holistic wellbeing that makes sense of the idea that ageing with health is also living with purpose (Seligman, 2011; Alimujiang et al., 2019).

### 4.2 Predictive power of the profiles

This work not only allows us to identify clinically differentiated profiles that can guide more tailored preventive strategies, but also provides evidence on the discriminative capacity of these profiles in key variables such as suicidal ideation. The High Risk profile showed significantly higher scores than the other profiles in terms of risk variables, thus validating its usefulness as a clinically prioritised group requiring immediate action (tertiary prevention). Conversely, the Optimal Mental Health profile exhibited the lowest levels of suicidal ideation, necessitating universal prevention measures and well-being promotion (primary prevention). This profile significantly diverged from the intermediate Burdensomeness and Weakened Personal Strengths profiles, for which secondary and selective prevention measures (individual or group) would be necessary.

This approach does not replace clinical assessments or crisis protocols; rather, it complements them by offering a more understandable and applicable view in contexts where specialised training is limited, such as in many nursing homes (Redondo et al., 2025). Unlike traditional screening, which focuses on current ideation, profiles enable the identification of psychological patterns that predispose individuals to varying degrees of vulnerability. For instance, an individual may not express active suicidal thoughts, yet exhibit a severely impaired psychological profile characterised by low resilience, low purpose and high hopelessness, representing a ‘silent’ risk that conventional questionnaires might overlook.

Although the psychological profiles retain their explanatory power beyond sociodemographic variables, the ANCOVA analyses showed that both sex and perceived health influence overall levels of suicidal ideation. Consistent with recent reviews, evidence indicates that men exhibit higher rates of suicide mortality and lower emotional expression, whereas women tend to report greater suicidal ideation and affective symptomatology (Shelef, 2021; Connors et al., 2025). Additionally, although research specifically addressing self-rated health is limited, multiple studies concur that physical and cognitive decline increases vulnerability to suicidal thoughts and behaviours (Chauliac et al., 2020; Beghi et al., 2021).

From a health psychology perspective, it is crucial to understand the psychological profiles of older people living in nursing homes in order to develop effective, person-centred interventions that promote mental health and prevent suicidal thoughts. By identifying distinct patterns of risk and protective factors, this study provides a useful framework for designing preventive strategies that transcend clinical diagnosis and integrate psychosocial dimensions such as resilience, self-efficacy and purpose in life (Ki et al., 2024). These findings underline the need to address mental health in a holistic manner in older age, especially in residential settings where psychological vulnerability may go unnoticed.

### 4.3 Limitations

A limitation of this study is that the LPA requires the selection of a limited number of variables, which implies that some relevant aspects of mental health have been left out of the model, such as loneliness, anxiety, coping or spirituality. Moreover, although suicidal ideation has been considered as one of the key variables, passive suicide, a particularly frequent manifestation in residential settings (Lee, 2023), has not been explicitly included, and which could provide valuable information for a better characterisation of risk profiles.

Additionally, it should be acknowledged that the profiles obtained largely refer to participants without cognitive impairment and with full ability to respond to all questionnaires during the interview, as only a small proportion of the sample presented MCI. Therefore, future replications should be conducted exclusively with participants without cognitive impairment.

Finally, the cross-sectional design prevents us from establishing causal relationships between the variables analysed and the profiles identified. Future research should consider longitudinal designs to examine the evolution of the profiles over time and the effectiveness of personalised interventions based on these profiles.

## Data Availability

The raw data supporting the conclusions of this article will be made available by the authors, without undue reservation.

## References

[ref1] Alimujiang A. Wiensch A. Boss J. Fleischer N. L. Mondul A. M. McLean K. . (2019). Association between life purpose and mortality among US adults older than 50 years. JAMA Netw. Open 2:e194270. doi: 10.1001/jamanetworkopen.2019.4270, 31125099 PMC6632139

[ref2] Baessler J. Schwarcer R. (1996). Evaluación de la autoeficacia: Adaptación española de la escala de Autoeficacia General. Ansiedad Estrés 2, 1–8.

[ref3] Bareeqa S. B. Samar S. S. Masood Y. Husain M. M. (2023). Prevalence of suicidal behaviors in residents of long-term care facilities: a systematic review and meta-analysis. OMEGA 92, 83–102. doi: 10.1177/00302228231176309, 37247610

[ref4] Beach V. L. Brown S. L. Cukrowicz K. C. (2021). Examining the relations between hopelessness, thwarted interpersonal needs, and passive suicide ideation among older adults: does meaning in life matter? Aging Ment. Health 25, 1759–1767. doi: 10.1080/13607863.2020.1855102, 33317336

[ref5] Beck A. T. Kovacs M. Weissman A. (1979). Assessment of suicidal intention: the scale for suicide ideation. J. Consult. Clin. Psychol. 47, 343–352. doi: 10.1037/0022-006X.47.2.343, 469082

[ref6] Beghi M. Butera E. Cerri C. G. Cornaggia C. M. Febbo F. Mollica A. . (2021). Suicidal behaviour in older age: a systematic review of risk factors associated with suicide attempts and completed suicides. Neurosci. Biobehav. Rev. 127, 193–211. doi: 10.1016/j.neubiorev.2021.04.011, 33878336

[ref7] Bernier S. Lapierre S. Desjardins S. (2020). Social interactions among older adults who wish for death. Clin. Gerontol. 43, 4–16. doi: 10.1080/07317115.2019.1672846, 31615349

[ref8] Bickford D. Morin R. T. Woodworth C. Verduzco E. Khan M. Burns E. . (2021). The relationship of frailty and disability with suicidal ideation in late life depression. Aging Ment. Health 25, 439–444. doi: 10.1080/13607863.2019.1698514, 31809584 PMC8931702

[ref9] Canal-Rivero M. Silva C. Obiols-Llandrich J. E. García-Bernal C. García-Sanchez C. Bustos-Cardona T. . (2022). Toward understanding of suicidality in a Spanish clinical population: validation of the European Spanish version of the interpersonal needs questionnaire. Psychopathology 55, 16–27. doi: 10.1159/000519792, 34963119 PMC8944174

[ref10] Celeux G. Soromenho G. (1996). An entropy criterion for assessing the number of clusters in a mixture model. J. Classif. 13, 195–212. doi: 10.1007/BF01246098

[ref11] Chauliac N. Leaune E. Gardette V. Poulet E. Duclos A. (2020). Suicide prevention interventions for older people in nursing homes and long-term care facilities: a systematic review. J. Geriatr. Psychiatry Neurol. 33, 307–315. doi: 10.1177/0891988719892343, 31840568

[ref12] Cizková J. Dostálová V. Bártová A. Holmerová I. Jelínková P. V. (2024). Care of older adults with mental illness in long-term care residential facilities: a scoping review. J. Am. Med. Dir. Assoc. 25:105218. doi: 10.1016/j.jamda.2024.105218 39155046

[ref13] Connors M. H. Draper B. Wand A. P. De Leo D. Reppermund S. (2025). Suicide and self-harm in older adults. Nat Rev Psychol 4, 440–456. doi: 10.1038/s44159-025-00454-w, 41391393

[ref14] Conwell Y. Van Orden K. Caine E. D. (2011). Suicide in older adults. Psychiatr. Clin. North Am. 34, 451–468. doi: 10.1016/j.psc.2011.02.002, 21536168 PMC3107573

[ref15] Cramer R. J. Tucker R. (2021). Improving the field’s understanding of suicide protective factors and translational suicide prevention initiatives. Int. J. Environ. Res. Public Health 18:1027. doi: 10.3390/ijerph18031027, 33503803 PMC7908249

[ref16] Cukrowicz K. C. Cheavens J. S. Van Orden K. A. Ragain R. M. Cook R. L. (2011). Perceived burdensomeness and suicide ideation in older adults. Psychol. Aging 26, 331–338. doi: 10.1037/a0021836, 21401264 PMC3699192

[ref17] De Brigard N. (2021). Relación Sentido de Vida en Personas con ideación y/o comportamiento suicida: Una revisión sistemática. Perspect. Metodol. 21, 1–22. doi: 10.18294/pm.2021.3431

[ref18] De Leo D. (2022). Late-life suicide in an aging world. Nat Aging 2, 7–12. doi: 10.1038/s43587-021-00160-1, 37118360

[ref19] Ferguson S. L. Moore G. W. Hull D. M. (2020). Finding latent groups in observed data: a primer on latent profile analysis in Mplus for applied researchers. Int. J. Behav. Dev. 44, 458–468. doi: 10.1177/01650254198817

[ref20] García-Alandete J. Martínez E. R. Sellés Nohales P. (2013). Estructura factorial y consistencia interna de una versión española del Purpose-In-Life Test. Univ. Psychol. 12, 517–530. doi: 10.11144/Javeriana.UPSY12-2.efci

[ref21] Gysin-Maillart A. Soravia L. Schwab S. (2020). Attempted suicide short intervention program influences coping among patients with a history of attempted suicide. J. Affect. Disord. 264, 393–399. doi: 10.1016/j.jad.2019.11.059, 31759660

[ref22] Hawton K. Lascelles K. Pitman A. Gilbert S. Silverman M. (2022). Assessment of suicide risk in mental health practice. Lancet Psychiatry 9, 922–928. doi: 10.1016/S2215-0366(22)00232-2, 35952701

[ref23] Heisel M. J. Flett G. L. (2022). Screening for suicide risk among older adults. Aging Ment. Health 26, 392–406. doi: 10.1080/13607863.2020.1857690, 33327729

[ref24] Heisel M. J. Moore S. L. Flett G. L. Norman R. M. G. Links P. S. Eynan R. . (2020). Meaning-centered men’s groups. Clin. Gerontol. 43, 76–94. doi: 10.1080/07317115.2019.1666443, 31671031

[ref25] Hernandez S. C. Overholser J. C. Philips K. L. Lavacot J. Stockmeier C. A. (2021). Suicide among older adults. Int. J. Psychiatry Med. 56, 408–421. doi: 10.1177/0091217420982387, 33322985

[ref26] Keefner T. P. Stenvig T. (2020). Rethinking suicide risk with a new generation of suicide theories. *Res theory*. Nurs. Pract. 34, 389–408. doi: 10.1891/RTNP-D-19-00128, 33199410

[ref27] Khazem L. R. Jahn D. R. Cukrowicz K. C. Anestis M. D. (2015). Physical disability and the interpersonal theory of suicide. Death Stud. 39, 641–646. doi: 10.1080/07481187.2015.1047061, 26079648

[ref28] Ki M. Lapierre S. Gim B. Hwang M. Kang M. Dargis L. . (2024). A systematic review of psychosocial protective factors against suicide and suicidality among older adults. Int. Psychogeriatr. 36, 346–370. doi: 10.1017/S104161022300443X, 38305360

[ref29] Klonsky E. D. May A. M. (2015). The three-step theory (3ST). Int. J. Cogn. Ther. 8, 114–129. doi: 10.1521/ijct.2015.8.2.114

[ref30] Klonsky E. D. Saffer B. Y. Bryan C. J. (2018). Ideation-to-action theories of suicide. Curr. Opin. Psychol. 22, 38–43. doi: 10.1016/j.copsyc.2017.07.020 30122276

[ref31] Lee S. (2023). Passive suicidal ideation in older adults from 12 European countries. J. Popul. Ageing 16, 137–154. doi: 10.1007/s12062-021-09350-6

[ref32] Liu Y. Lan D. Zhou Y. Tian H. Xiao J. Gan L. . (2024). Role of subjective well-being and resilience…. Geriatr. Nurs. 59, 418–425. doi: 10.1016/j.gerinurse.2024.07.041, 39141949

[ref33] Lobo A. Saz P. Marcos G. Día J. L. de la Cámara C. (1999). Revalidación y estandarización del MEC. Med. Clin. (Barc.) 112, 767–741. 10422057

[ref34] Malfent D. Wondrak T. Kapusta N. D. Sonneck G. (2010). Suicidal ideation…. Int. J. Geriatr. Psychiatry 25, 843–849. doi: 10.1002/gps.2426, 19946865

[ref35] Masyn K. E. (2013). “Latent class analysis and finite mixture modeling” in The Oxford handbook of quantitative methods. ed. Little T. (Oxford: OUP), 551–611.

[ref36] McPherson C. J. Wilson K. G. Murray M. A. (2007). Feeling like a burden to others. Palliat. Med. 21, 115–128. doi: 10.1177/0269216307076345, 17344260

[ref37] Muthén L. K. Muthén B. O. (2017). Mplus User’s Guide. 8th Edn. Los Angeles, CA: Muthén & Muthén.

[ref38] Nie Y. Hu Z. Zhu T. Xu H. (2020). Prevalence and risk factors for suicidal ideation among the elderly in nursing homes. Front. Psych. 11:339. doi: 10.3389/fpsyt.2020.00339, 32477170 PMC7241427

[ref39] O’Connor R. C. Kirtley O. J. (2018). The integrated motivational–volitional model of suicidal behaviour. Philos. Trans. R. Soc. Lond. Ser. B Biol. Sci. 373:20170268. doi: 10.1098/rstb.2017.0268, 30012735 PMC6053985

[ref40] Okolie C. Dennis M. Thomas E. John A. (2017). Interventions to prevent suicidal behaviors in older people. Int. Psychogeriatr. 29, 1801–1824. doi: 10.1017/S1041610217001430, 28766474

[ref41] Olatunji O. A. Idemudia E. S. Olawa B. D. (2020). Family support, self-efficacy and suicidal ideation…. Int. J. Adolesc. Youth 25, 920–931. doi: 10.1080/02673843.2020.1779762

[ref42] Ostafin B. D. Proulx T. (2020). Meaning in life and resilience to stressors. Anxiety Stress Coping 33, 603–622. doi: 10.1080/10615806.2020.1800655, 32755239

[ref43] Pinazo-Clapés C. Redondo R. Checa I. Pinazo-Hernandis S. Sales A. Pons J. (2025). Screening of suicidal ideation in nursing homes. Aging Ment. Health 1–8, 1–8. doi: 10.1080/13607863.2025.2545357, 40808588

[ref44] Redondo R. Pinazo-Clapés C. Checa I. Pinazo-Hernandis S. Sales A. (2025). Analysis of professionals' perceptions…. Int. J. Geriatr. Psychiatry 40:e70152. doi: 10.1002/gps.70152, 40910417 PMC12412084

[ref45] Ribeiro J. D. Huang X. Fox K. R. Franklin J. C. (2018). Depression and hopelessness as risk factors…. Br. J. Psychiatry 212, 279–286. doi: 10.1192/bjp.2018.27, 29587888

[ref46] Salvatore T. (2023). Dying by suicide in nursing homes. OMEGA 88, 20–37. doi: 10.1177/00302228211038798, 34404260

[ref47] Satorres E. Ros L. Meléndez J. C. Serrano J. P. Latorre J. M. Sales A. (2018). Measuring elderly people's quality of life through the Beck hopelessness scale. Aging Ment. Health 22, 239–244. doi: 10.1080/13607863.2016.1247427, 27786537

[ref48] Segal D. L. Marty M. A. Coolidge F. L. Armstrong M. (2025). Suicidal ideation in older adults…. Psychol. Rep.:00332941251340310. doi: 10.1177/00332941251340310 40324800

[ref49] Seligman M. E. P. (2011). Flourish. New York: Simon & Schuster.

[ref50] Shelef L. (2021). The gender paradox: do men differ from women in suicidal behavior? J. Men's Health 17, 22–29. doi: 10.31083/jomh.2021.099

[ref51] Shim Y. Choe K. Kim K. S. Kim J. S. Ha J. (2021). Applicability of the interpersonal–psychological theory of suicide among community-dwelling older persons. Suicide Life Threat. Behav. 51, 816–823. doi: 10.1111/sltb.12757, 33870547

[ref52] Sinclair V. G. Wallston K. A. (2004). Brief resilient coping scale. Assessment 11, 94–101. doi: 10.1177/1073191103258144, 14994958

[ref53] Soucase B. García-Alandete J. Rubio-Belmonte C. (2023). Presence/search for meaning and positive functioning. Curr. Psychol. 42, 2198–2207. doi: 10.1007/s12144-021-02394-z

[ref54] Tein J. Y. Coxe S. Cham H. (2013). Statistical power to detect the correct number of classes in latent profile analysis. Struct. Equ. Modeling 20, 640–657. doi: 10.1080/10705511.2013.824781, 24489457 PMC3904803

[ref55] Treichler E. B. Glorioso D. Lee E. E. Wu T. C. Tu X. M. Daly R. . (2020). Group intervention to increase resilience in senior housing. Int. Psychogeriatr. 32, 173–182. doi: 10.1017/S1041610219002096, 32017867 PMC7526871

[ref56] Van Orden K. A. Bamonti P. M. King D. A. Duberstein P. R. (2012). Does perceived burdensomeness erode meaning in life among older adults? Aging Ment. Health 16, 855–860. doi: 10.1080/13607863.2012.657156, 22401290 PMC3416966

[ref57] Van Orden K. A. Witte T. K. Cukrowicz K. C. Braithwaite S. R. Selby E. A. Joiner T. E. Jr. (2010). The interpersonal theory of suicide. Psychol. Rev. 117, 575–600. doi: 10.1037/a0018697, 20438238 PMC3130348

[ref58] Vilhjálmsson R. Sveinbjarnardottir E. Kristjansdottir G. (1998). Factors associated with suicide ideation in adults. Soc. Psychiatry Psychiatr. Epidemiol. 33, 97–103. doi: 10.1007/s001270050028, 9540383

[ref59] WHO (2020). Decade of healthy ageing 2020–2030. Geneva: WHO.

[ref60] WHO (2024). Mental health: Strengthening our response. Geneva: WHO.

[ref61] Xu D. Wang Y. Li M. Zhao M. Yang Z. Wang K. (2022). Depressive symptoms and ageism among nursing home residents. Int. J. Environ. Res. Public Health 19:12105. doi: 10.3390/ijerph191912105, 36231405 PMC9564776

[ref62] Yang Y. Wang R. Zhang D. Zhao X. Su Y. (2021). How loneliness worked on suicidal ideation among Chinese nursing home residents. Int. J. Environ. Res. Public Health 18:5472. doi: 10.3390/ijerph18105472, 34065364 PMC8160705

[ref63] Yesavage J. A. Brink T. L. Rose T. L. Lum O. Huang V. Adey M. . (1983). Development and validation of a geriatric depression screening scale. J. Psychiatr. Res. 17, 37–49. doi: 10.1016/0022-3956(82)90033-4, 7183759

[ref64] Yuan Y. Barooah A. Lapane K. L. Mack D. Rothschild A. J. Ulbricht C. M. (2022). Health profiles of older nursing home residents by suicidal ideation. Int. J. Geriatr. Psychiatry 37. doi: 10.1002/gps.5829 PMC10165533 36281640

[ref65] Zhang D. Wang R. Zhao X. Zhang J. Jia J. Su Y. . (2021). Resilience and social support in the relationship between loneliness and suicidal ideation. Aging Ment. Health 25, 1262–1272. doi: 10.1080/13607863.2020.1786798, 32602736

